# Healthy sleep score, acute myocardial infarction, and type 2 diabetes

**DOI:** 10.1111/1753-0407.70019

**Published:** 2024-10-24

**Authors:** Tomoyuki Kawada

**Affiliations:** ^1^ Department of Hygiene and Public Health Nippon Medical School Tokyo Japan

Du et al.^1^ conducted a prospective study to investigate the effect of healthy sleep pattern on subsequent mortality risk of acute myocardial infarction (AMI) in people with diabetes. The adjusted hazard ratio (HR) (95% confidence interval [CI]) of healthy sleep score for AMI mortality was 0.87 (0.77–0.98). Especially, adequate sleep duration reduced 29% risk of AMI mortality. They finally mentioned that types of diabetes should be stratified for the analysis, and I think that interactions of diabetes on the inverse association between healthy sleep score and AMI mortality may be existed. In their Figure 1, there is a wide range of HR for the risk of AMI mortality in lower healthy sleep score, which did not reach a significant level. There is a possibility that people with lower healthy sleep score would have several cardiometabolic risk factors, and contribution rate of sleep variables to the risk of AMI would become smaller. I speculate that irregular sleep pattern in daily life reflects one of the unhealthy lifestyles, and it would contribute to diabetes and AMI risk. I present recent reports on the association between irregular sleep and subsequent risk of type 2 diabetes (T2D) or cardiometabolic disorder.

Zuraikat et al.^2^ defined irregular sleep pattern as the standard deviation of each sleep parameter, and it was closely related to the increased risk of T2D and cardiometabolic risk. Although causal association cannot be determined, irregular sleep pattern may contribute to the risk of several metabolic disorders.

Liu et al.^3^ conducted a meta‐analysis on the relationship between daytime napping and incident diabetes, and longer period of napping should be avoided to reduce a risk of diabetes. I suppose that long napping would affect nighttime sleep depth and duration, which may also relate to irregular sleep pattern.

Zhang and Qin^4^ reported the potential mechanisms regarding the effect of irregular sleep pattern on subsequent cardiometabolic risk, including circadian dysfunction, inflammation, autonomic dysfunction, endocrinological disorder, and gut dysbiosis. Glucose metabolism may be affected by unstable sleep–wake cycle and their duration, which would be closely related to the level of physical activity and nutritional intake.

Finally, Zhu et al.^5^ reviewed and concluded that sleep variability was significantly associated with weight gain and increased hemoglobin A1c, although decreased insulin sensitivity was not consistent findings in several studies.

## FUNDING INFORMATION

There is no financial support for this study.

## CONFLICT OF INTEREST STATEMENT

The author declares that he has no competing interests. No ethical statement is needed for this study.
